# Characteristics and components of children’s and adolescents’ resilience in disasters in Iran: a qualitative study

**DOI:** 10.1080/17482631.2018.1479584

**Published:** 2018-06-22

**Authors:** Leila Mohammadinia, Davoud Khorasani-Zavareh, Abbas Ebadi, Hossein Malekafzali, Ali Ardalan, Mojtaba Fazel

**Affiliations:** a Department of Disaster Public Health, School of Public Health, Tehran University of Medical Sciences, Tehran, Iran; b Health Human Resource Research Center, Department of Health in Disasters and Emergencies, School of Management &Information Sciences, Shiraz University of Medical Sciences, Shiraz, Iran; c Safety Promotion and Injury Prevention Research Center, Shahid Beheshti University of Medical Sciences, Tehran, Iran; d Department of Health in Disaster and Emergency, School of Health, Safety and Environment, Shahid Beheshti University of Medical Sciences, Tehran, Iran; e Department of Clinical Science and Education, Södersjukhuset, Karolinska Institutet, Stockholm, Sweden; f Behavioral Sciences Research Center, Life Style Institute, Faculty of Nursing, Baqiyatallah University of Medical Sciences, Tehran, Iran; g Department of Heath Policy, Permanent Member of Iranian Academy of Medical Sciences, Tehran, Iran; h Harvard Humanitarian Initiative, Harvard University, Cambridge, MA, USA; i Pediatric Nephrology, Valiasr Hospital, Imam Complex, Tehran University of Medical Science, Tehran, Iran

**Keywords:** Resilience, disasters, children and adolescents, qualitative study, Iran

## Abstract

Children and adolescents are vulnerable in times of disaster and they will suffer more severely if neglected. The concept of resilience differs between cultures, and identifying the components of resilience is essential for decision making and interventions in disasters such as risk management. This study aimed to identify the components of children’s resilience in disasters in Iran. This qualitative study took a content-analysis approach. Data were collected through semi-structured interviews with 23 people and three group meetings. Conventional content analysis was used for data analysis. MAXQDA 10 software was used for classification. The resilience components derived from the data were categorized into two main categories, internal and external, and eight subcategories covering psychological, emotional, cognitive, mental, spiritual, physical, social, and behavioral factors. The results also showed that the nature of resilience is both intrinsic and extrinsic. Recognizing the dimensions of children’s resilience in disasters can lead to a new perspective for authorities and planners in disaster and emergency situations. The results of this study could be used by planners and policymakers to develop interventions to enhance children’s and adolescents’ resilience at the time of disasters, which is also underlined and highlighted by international documents.

## Introduction

Disasters threaten the rights and lives of millions of children in the world, especially in low- and middle-income countries (Martin, 2010; Mudavanhu et al., 2015). According to the World Report in 2011, about 66 million children are affected by disasters annually (Mudavanhu et al., 2015), and disasters play an important role in the development of children’s personality and psychology (Feitelberg, 2007). To enhance children’s understanding and promote their readiness for disasters, their views could be used as a strategy in disaster management programmes. Given the importance of children’s views, the new document “Framework for Disaster Risk Reduction 2015–2030 (SFDRR)” suggests that children’s capacity should be used in disaster risk-reduction programmes (Cumiskey, Hoang, Suzuki, Pettigrew, & Herrgard, 2015). Children have the ability and capacity to reduce the risks of disaster in society (Mohammadinia, Khorasani-Zavareh, & Ardalan, 2016). Identifying and using the capacities and characteristics of children can increase the resilience of their society in disaster situations.

The resilience of children can grow in a positive way, in theory, and this is noticeable when they are faced with a health hazard more than at other times, because in disasters, the environment, community, and individual factors are impaired (Zimmerman et al., 2013). In disasters, parents may not have enough time and energy to care their children, and in these stressful situations, some children can adapt to the new situation, stay quite well, have fun, and even help others. Moreover, children can actively participate in interventional programmes and show positive and effective behaviours to improve their ability to reverse the adverse changes caused by the disaster (Hestyanti, 2006).

The focus on positive psychology has been increasing since the 1970s, with the aim of facing difficulties positively and effectively, and relying on the strengths of individuals rather than their weaknesses (Phuphaibul, Thanooruk, Leucha, Sirapo-Ngam, & Kanobdee, 2005). Most studies agree that resilience is a complex and a context-dependent concept (Connor & Davidson, 2003; Masten & Obradović, 2006). Resilience depends on the cultural context of a society, making the measurement of resilience a challenging task. This has encouraged many scholars to conduct research in this area (Masten, Powell, & Luthar, 2003; Rutter, 1987). Experts have various opinions about the nature of resilience, with some believing that resilience is intrinsic and others that it is extrinsic (Cheraghi, Ebadi, Gartland, Ghaedi, & Fomani, 2016; Martinez Garcia & Sheehan, 2016; Sapienza & Masten, 2011; Ungar, 2015). But, in general, resilience is an intrinsic–extrinsic concept and one of the most important components affecting health (Zolkoski & Bullock, 2012). Resilience as a personal characteristic leads to positive adaptation and moderates the negative effects of stressors, enabling people to rehabilitate and maintain their health in spite of existing problems (Tonmyr, Wekerle, Zangeneh, & Fallon, 2011; Wagnild & Young, 1993). According to Zolkoski and Bullock (2012), studies conducted over the past four decades on the concept of resilience indicate that this is a challenging concept for researchers, and there are several different definitions of resilience (Jacelon, 1997; Leipold & Greve, 2009; Nourian, Mohammadi Shahbolaghi, Nourozi Tabrizi, Rassouli, & Biglarrian, 2016; Vinson, 2002).

A previous study indicated that the recognition and understanding of the concept of resilience in the context of Iranian culture can help health planners to set health programmes for adolescents, identify their needs, and take effective measures to prevent injury and promote their resilience (Nourian et al., 2016). Studies conducted on resilience in Iran have been mainly focused on the areas of psychology and psychiatry, describing the relationship between resilience and the psychological, familial, occupational, and social characteristics of adolescents of different ages. Although studies on resilience have been increasing in recent years, few studies have been conducted to examine the components of children’s resilience in disasters (Mohammadinia et al., 2016, 2017). For example, the study by de Milliano (2015) divided the self-resilience model into three internal components (cognitive, behavioural, and spiritual) and four external components (social, political, economic, and environmental) (de Milliano, 2015). However, considering the environmental degradation and the disappearance of economic and political infrastructure in disasters, it cannot be said that children’s resilience is dependent on economic and political support. Despite extensive research on adolescents’ resilience, there is no comprehensive and transparent definition of the components of children’s and adolescents’ resilience (Ahern, Kiehl, Lou Sole, & Byers, 2006; Lock & Janas, 2002; Mandleco, 2000).

Disasters and emergencies affect the lives of many children each year, and Iran is a country with high rates of natural and human-made hazards. Since researchers agree that childhood is the best time to create resilience (Lock & Janas, 2002), it is necessary to take into account the potential and capacities of children’s resilience to reduce the risk of disaster. As the concept of resilience is abstract, health planners and policymakers need to elucidate the objective aspects and components of children’s resilience in disasters. Therefore, this qualitative study was designed to identify the characteristics and components of children’s resilience in natural disasters.

## Material and methods

This qualitative study was conducted using a conventional content-analysis approach.

### Study participants and data collection

In total, 23 experts in health and policymaking, health management, and child psychology, as well as teachers and senior executives in the field of disaster management, participated in this study. All interviews were conducted in Tehran. The purposeful interviews were conducted between September 2016 and July 2017. Inclusion criteria for interviewees were having experience in disasters and incidents as a manager, or experience working with children, or both. The individual interviews lasted between 20 and 120 min, with an average of 46 min. The interviews started with semi-structured questions developed by the research team. Different questions were asked to identify the children’s resilience and capacities, including “Please describe your experience of past disasters in which you have been in contact with children.” “Have there been any children who were different from others, either helping or undertaking specific action?” “What capacities do you think the children have to help in disasters?” “If you are familiar with the concept of resilience, what kind of child do you call resilient?” “What are the characteristics of children who face problems caused by disasters and do not give in?” “What do you think about these children?” “Do you know or remember any child who has these characteristics?” According to the data requirements, the questions could change for different participants.

In addition, 23 face-to-face interviews, three expert panel meetings with disaster management and children’s institutions, including the Disaster Prevention and Management Organization, Red Crescent, Ministry of Health and Medical Education, United Nations Children’s Fund (UNICEF), Welfare Organization, Municipality, and Education, were held to clarify the components of children’s resilience in disasters. At the first meeting, all deputies or representatives of the aforementioned organizations were present, and the next two sessions of the meeting were held with the organizations’ experts in the field of disasters.

In terms of demographics, among the 23 interviewees, one was a 16-year-old female student, and 22 interviewees were experts and specialists in the fields of health, psychology, and disaster management with an age range of 36–73 years and a mean age of 48 years. All participants had more than 5 years of work experience in the area of disasters and emergencies. Considering the numerous definitions of children, at the beginning of the interview, the main researcher outlined the definition of children based on the UNICEF/United Nations’ definition that refers to a child as any person who is less than 18 years old (Erskine et al., 2017). In addition, the researcher explained the purpose of the study, which was to identify the characteristics and components of children’s resilience in disasters for the 12–18-year-old age group.

### Data analysis

After each interview, the audio file was implemented accurately by LM. Open-source coding with a word-by-word and paragraph-by-paragraph combined approach was immediately applied and the codes were evaluated by DKZ, an expert in the qualitative field. If correction was needed, re-encoding was carried out. The coding process was performed as meaning units, condensed meaning units, and then codes. Finally, categories and subcategories were formed by LM, HM, and AE. Different codes were compared and categorized based on their similarities and differences. Then, the codes were analysed and categorized to produce the main categories, and the qualitative table of components was extracted by determining the domains.

### Rigour

To validate the research, a summary of the interview was returned to participants after implementation, to confirm the accuracy of the interview. In addition, the codes were monitored during the coding process and evaluated by the qualitative expert and the corresponding author. To identify the components of children’s resilience in disasters, after the researchers had listened to the audio files several times and spent a long time analysing and interpreting the data, MAXQDA 10 software was used for categorization and quotations. After summarizing and categorizing the codes, several sessions were designated for peer review, and the categorization table was repeatedly corrected. The coding and research processes were supervised by an expert in qualitative study. The most time was devoted to extracting codes, with different experts having different perspectives. Therefore, frequent meetings were held to establish logical relationships between codes and categories. In addition, to cover the issue of resilience, the perspectives of specialists in different fields, including psychology, disaster management, paediatric nursing, and policymaking, as well as teachers and students, were considered carefully.

### Ethical considerations

The interview date was coordinated by telephone. The interview place was based on the interviewee’s request. At the beginning of the interview, the interviewer explained the purpose of the study and, once permission had been received to record the interview, participants were informed about the principles of confidentiality and anonymity of the interview.

This research is part of the health in disaster and emergency PhD thesis at the Faculty of Public Health, Tehran University of Medical Sciences, which has received ethical approval from the Ethics Committee of Tehran University of Medical Sciences (code IR.TUMS.SPH.REC.1395.1542).

## Results

Of the interviewees, 30% were female, and they had often been educated to the master’s, PhD, or post-doctoral level (Table I). All interviews were conducted by the principal investigator. The 23 face-to-face interviews and three specialized meeting sessions were held and resulted in 416 primary codes for children’s capacity in disasters. The extracted codes were reviewed, merged, and classified into two main categories of internal components, including psychological, emotional, cognitive, mental, spiritual, and physical components; and external components, including social and behavioural skills (Table II).

**Table I.** Participants’ characteristics in the child resilience domain in disasters, Iran, 2016–2017.

Experience	Educational level	Age (years)	Gender
Architecture, urban conservation, reconstruction	Postdoc: 9%	Range: 36–73	Male: 70%
International Federation of the Red Cross	PhD: 68%	Mean: 48	Female: 30%
Emergency medicine specialist	MS: 13%		
University professor, Director of Health Department in Disasters	BS: 5%		
Tehran Disaster Mitigation and Management Organization	MS: 13% to 18 %		
University professor, health policy, community health			
Postdoctoral psychologist			
Child psychologist			
Nurse with 15 years’ experience in disasters			
Deputy Director General for Healthcare, Red Crescent Society			
University professor, child nursing			
University professor, Head of the Department of Psychology at the Welfare Organization			
Psychologist, Mental Health Department, Ministry of Health			
Schoolteacher with 24 years’ experience			
Professor, Director of the Department of Health in Disasters, Risk Specialist			
Executive Director, Moderator, Red Crescent Organization			
Professor of paediatrics

**Table II.** Components of resilient children in disasters in Iranian society: qualitative content analysis results, 2017.

Theme	Subtheme	Codes
Internal	Psychological, emotional	Living in the moment Being energetic Having motivation Power of compromise and compatibility Being honest Showing emotion easily Fast emotional return Desire to be seen
	Cognitive, mental	Proving themselves to others Looking to the future and optimism Not having prejudices about issues Coping with problems quickly Being free from rigid rule and policy Having a strong mind and good memory Searching and curious mind Continues learning and never forget High-speed learning Interested in learning Problem-solving skills Creative thinking High adaptability to environmental conditions No marginality or bias
	Spiritual	Having a pure spirit and soul Sense of friendship Not having material dependency Belief in support of a supernatural force (trust in God) Worship, learning and reading the holy books, performing rituals and attending religious ceremonies, self-confidence, relying on belief in God
	Physical	High-speed physical activity Active and dynamic Physically fast
External	Social skills	Interested in playing a social role Teamwork ability Children’s acceptance and popularity Primary ring of family formation Factor of happiness and happy family Factor of influencing changes in family and society
	Behavioural skills	Hard work Independence Ability to support younger children Accepting and following commands Voluntary contributions to work without expectation Potential of change behaviour Self-control and other-control More resilient communication than adults Having the influence of the word Transferring knowledge to others Flexibility Providing services to family in disasters Being alert for others

### Internal components

According to participants in this study, the internal components included psychological, emotional, cognitive, mental, spiritual, and physical components. The internal domains cover the most important and broadest components of children’s resilience and capacities in disasters.

#### Psychological components

The results of this study showed that resilient children have a rapid mental recovery and can easily criticize others fairly. They also have characteristics such as self-care, self-esteem, self-confidence, and lack of attention to previous concerns. They can speak honestly and have a lot of motivation and energy when performing a task. They try to improve the conditions caused by disasters, and, unlike adults, they have an optimistic view about issues and try to be positive at all times. Children’s remarkable memory and intellectual capacity increase their learning speed and their curiosity in education, creativity, and problem solving.

Children helped their illiterate neighbours to do their work like borrowing a loan. For instance, a child said, my neighbour wanted to go to a certain place and I went with him and filled out a form. Well, this shows that they had the manner of helping others, and I do not know whether the sense of altruism has been strengthened in him. Now in a disaster situation, instead of just paying attention to himself or his family, he helps others as well. (E.11)

Children were also very comfortable moving the sick patients, caring for people, and comforting and visiting them. (E.5)

#### Cognitive and mental components

These components related to children’s mental ability and their way of thinking about themselves and their life. They are related to some subjective criteria such as proving themselves to others, looking to the future and optimism, not having prejudices about issues, coping with problems quickly, Being free from rigid rules and policy, having a strong mind and good memory, having a searching and curious mind, high-speed learning, being interested in learning, problem-solving skills, creative thinking, and being highly adaptable to environmental conditions.

Children will recognize problems very soon, which means they will solve the issues very soon for themselves and return to the state of happiness and the period of childhood, well, in my opinion, this shifting a child from an accident to a normal life is a very good model in the disaster. (E.15)

#### Spiritual component

The spiritual component, sometimes called spiritual well-being, is a component of children’s capacity, which if promoted, can increase the children’s resilience in disasters. The spiritual components of children’s resilience in disasters include their pure feelings and spirit, such as empathy towards other children and adults, and their kindness in dealing with others. Children, unlike adults, have no material dependency and pursue their pure intrinsic power. Examples of this include believing in the support of a superior power (trusting in God), obtaining peace and energy through worship, reading the holy books, attending religious ceremonies and performing rituals, and increasing self-esteem by relying on God. The spiritual component can also promote the psychological component. Believing in a divine power causes children to be hopeful and reduces their mental problems, especially during times of difficulty and challenges, as in disasters. Having a spiritual belief can act as a driving force for children.

… This is resilience when they say that, I will make the will of God my own will, which means excellence, positive thinking, and hope for the future. (E.13)

… Resilience is not joy, or optimism, or surrendering to the mercy of God, the resilience is thinking. What does that child say? He says that everyone has a destiny and that is God’s will. What have I come here to do? I pray for my mom every night who has died, I read Ayat-Al Korsi [a verse in the Quran] so my mom can be blessed. Here, this child is becoming resilient …. (E.13)

#### Physical component

The physical component was another element of children’s resilience. Children’s abilities included fast physical activity and being active, faster physical recovery, and higher physical stamina. The activity, vigour, energy, and dynamism of children come from their physical energy, which brings joy and vitality to the society and family. This can be considered as a capacity in children that increases their readiness and reduces vulnerability at a time of accidents and disasters, which is the same as the children’s physical resilience.

Children have a higher level of health than adults and have higher hopes for the future. (E.5)

### External components

External components included social and behavioural skills that determine the children’s resilience, when interacting with their environment.

#### Social skills

Although the social component covers an independent domain, it has a close relationship with the psychological component. In this study, examples of social skills as the social capacity of children included having a spirit of social acceptance and the ability to work in a group, better coordination, an interest in taking a social role, and a participatory spirit. Children have the ability to attract the support of others and influence people. They are distanced from the everyday activities and mental engagements that exist in adults and have negative impacts on social activities. Moreover, they give hope to the family and act as a stimulus to improve the conditions of society and return it to the pre-disaster situation. Children can also help to reduce the risk of disasters by transferring knowledge and lessons learned (about the risk of disasters and actions that need to be taken) to families and communities. Older children and adolescents can inform the neighbourhood, give alerts, help with the distribution of goods, participate in the psychological and social reconstruction of the society, and return the community to its pre-disaster state, not only in the prevention phase of disaster management, but also in the phases of response and recovery.

Children’s participation in social programmes, especially following a disaster, can not only serve as a human resource to help improve the conditions and return them to normal more quickly, but also prevent the children being passive and withdrawn, and recalling bad memories caused by accidents and disasters.

… Something that I experienced myself in the Bam earthquake, when children are given a responsibility, they recover faster. For example, I told a 15-year-old kid; you’re responsible for going to Bravat district and identifying any families who need a water tank, and come back and tell us so we can supply them with a water tank; this gave him an identity and he felt like he was being seen. He was going to different places, and was bringing us information about which family had a water tank and which one did not. This is an example of how they should be given a responsibility which is proportionate to their age. (E.4)

Learning from the community and face-to-face education in schools also has an important role in promoting children’s resilience and the transfer of disaster knowledge to children’s families. Some experts had concrete examples in this regard. One of the professors living in the Darabad Tower said that:

It was Friday afternoon when the earthquake happened, my wife ran in one direction and I ran in another direction. My 5-year-old daughter ran towards the door and opened her arms and legs and said, “Mom, Daddy why are you scared? Auntie told me, at the time of an earthquake when you are at home, you should stand under the door frame and open your hands and legs”. We saw this kid had taught us, and we are architect professors!! She said, “Do not be scared”. (E.1)

#### Behavioural skills

Children’s behavioural components are among other indicators that reflect their resilience when interacting with others and during their interactions with their surroundings. Resilient children have special behavioural skills; for example, they support younger children. In fact, since resilient children would like to be heroes, they work really hard. They voluntarily help and follow commands without expectation. They undertake the task given to them, and one can be sure that the work will be done.

Children take responsibility better than elders. If you ask a child to do homework, he does not stop until the homework is done, but if you ask an adult to do something, he will make thousands of excuses not to do it. For this reason, if we assign a task to children, we can be sure the task will be done if they understand the task. For instance homework; you rarely see a child that has not done his homework and makes excuses. (E.5)

Children in disaster situations not only control their childish behaviours to help the situation return to normal, but also, by reminding each other and controlling other children, try to bring calm to their own society.

For example, as children appear in foreign films, the devotees try to observe the rules and for a while, even the guilty children think, “If we do this, the situation will return to normal sooner”, that is, they will control it with this thinking. One of the behaviours that is seen in children after disasters is destructive behaviours, breaking glass, for example, but there are some children who say, “Do not do this, why do you do this?” There is potential within them, that is, to monitor that others do not make a mistake. (E.14)

Another behavioural skill of resilient children is that they communicate well with others, including friends and other adults, so it can be said that they have better communicational resilience than older adults. A sense of humour, behavioural flexibility, and a desire to be heroic and courageous are some of the other components that are categorized in the field of behavioural skills in this study.

… They try to help their parents with everyday housekeeping duties and outside work; for example, helping their parents with borrowing loans and so on. There were children who were helping their illiterate neighbours with work such as borrowing loans. (E.11)

Because of the children’s flexibility, they can easily model and change their behaviour to a large extent. This is an important issue that needs to be addressed in times of disaster. Behavioural skills can also be taught and transferred to children with appropriate instructional education. The good spirits and honest behaviour of children have a profound emotional impact on society.

… For example, I remember the children under the age of 18, who were coming to help with clean spirit and good intentions. They were undertaking large tasks without expectation. They were very influential and we had no problem with them. (E.4)

## Discussion

Although the nature of resilience has been discussed for years, its application in the field of disasters is still quite new. Children’s resilience in disasters is a complex and challenging concept. This study showed that children have significant characteristics and capabilities in disaster risk reduction. Considering that most studies have pointed to the vulnerability of children in disasters, eight components of psychological, emotional, cognitive, mental, spiritual, and physical, as well as social and behavioural skills in regard to children’s resilience in disasters were extracted from the data in this study. It should be mentioned that as some components were very close to each other, the research team preferred to integrate psychological and emotional criteria into one group, and cognitive and mental criteria into another. Therefore, as mentioned in Table II, the subjective examples have been summarized in six domains for greater clarity.

The psychological, emotional, cognitive, and mental components cover a broad and important area of children’s resilience in disasters. As this study showed, there are many indicators in this area. In line with this finding, other studies have shown that mental health is one of the most important components of children’s resilience in disasters that most scholars have emphasized. Recent studies have also emphasized the importance of the psychological component as a factor that affects children’s resilience (Fu, 2012; Grotberg, 2001; Massad et al., 2009; Masten, 2014; Wang, Zhang, & Zimmerman, 2015). Other studies have identified psychological, emotional, cognitive, and mental components as individual and personality traits or characteristics (Jacelon, 1997; Wagnild & Young, 1993), which can determine the capacity of children in disasters, in line with the current study. These characteristics can also be used as indicators of children’s resilience. The psychological components are so significant that Greenberg (2006) considers emotional management to be one of the most important criteria of resilience, with the importance of this issue becoming evident during and after disasters. Not only in normal situations, but also in disasters, this component helps to control children’s destructive behaviour in their interaction with each other, their parents, and society. In the challenging environments of post-disaster camps, it is very helpful to strengthen children’s emotional spirit as a life skill, which will also be very beneficial for their future.

One of the children’s qualities is their view of the future. Since children are less involved in life issues than adults, this can increase their creativity and vitality. So, in the event of a disaster, children can give hope to the family and society, and enable society to return more quickly to normal conditions. The popularity of children can influence others to recover from disaster situations. Along with the results of this study about children’s optimistic views, Fredrickson (2004) also noted that the role of positive emotions in responding to events is very prominent, because these feelings can make a person change their mind instantly and seek personal resources, and prepare them, both behaviourally and mentally, for future events. Self-esteem (Zimmerman & Ringle, 1981) and problem-solving skills (Williams & Krane, 1992) as psychological concepts are other personal components that are in line with the results of this study, which can lead to effective interventions by children in disaster situations.

Spirituality is one of the factors in this study that influenced the resilience of children at the time of the disaster. Communication with the creator, worship, and prayer, in different cultures and different religions, has calming effects, such as improved mental health and increased tolerance. Other studies have also indicated the effects of a spiritual component on the resilience of children and adolescents (de Milliano, 2015; Nourian et al., 2016; Sapienza & Masten, 2011). Kindness and empathy make up another component of children’s resilience, which in disasters can be effective in promoting the mental and psychological condition of both the society and the family. As Clarken (2009) stated, children who have more empathy are kinder than others and show more caring behaviours. These children are sensitive and worry about others being harmed (Clarken, 2009). High levels of empathy and tolerance influence the resilience of most young people, and empower them to communicate better with their own circle, other people, and society, despite their differences (Gartland, Bond, Olsson, Buzwell, & Sawyer, 2011). In line with the findings of the present study, children are often the first to help others and these efforts not only help them to meet their psychological needs, but also reduce their vulnerability to accidents and disasters, according to experts.

The personal components and aforementioned characteristics, alongside the children’s social and behavioural components, form resilience in children in disaster situations. The internal and external components are not separate from each other and their boundaries sometimes overlap. With regard to the social and behavioural components, Zautra (2013) believes that resilience is based on social foundations, and although individuals have internal capacities to confront tensions, the appearance of resilience requires an appropriate situation with proper communication and social interactions. According to Ananiadou and Claro (2009), social skills are considered as desirable competencies for life in the twenty-first century, and are a set of skills that is used for communication with the people in one’s environment. One of these skills is interpersonal relationships (Ananiadou & Claro, 2009). According to the results of our study, resilient children have good communication skills and can be a great help to themselves and others in disaster situations through proper interactions. They can also team up with peers through proper coordination and help older people and younger children who have been affected by the disaster.

Other studies have identified six components for social skills, namely the ability to influence others, conflict resolution, leadership and the ability to facilitate the process of change, establishing emotional bonds with others, collaborative work, and teamwork and effective communication with team members (Ishak, Abidin, & Bakar, 2014). In the present study, interactions in the family and the role of parents in enhancing children’s resilience were significant because children’s behaviour and learning are shaped by the family to a large extent. Other researchers have recognized the role of family in helping adolescents to adapt to tensions or unpredictable problems during adolescence (Cridland, Jones, Magee, & Caputi, 2014; Tatnell, Kelada, Hasking, & Martin, 2014).

In addition to the family, other supporting environments, including the school and its supporting role, have a significant impact on the promotion of children’s resilience. In other words, with regard to the environmental components, social supports such as family, friends, and the school environment have been confirmed as factors that affect adolescents’ resilience (Dray et al., 2015; Hunter, 2001; Tiet et al., 1998), which is consistent with the results of the present study. Some countries, such as the UK, have formulated and implemented programmes aimed at promoting children’s resilience based on the role of the school (Challen, Machin, & Gillham, 2014). However, in a number of studies, no relationship has been found between resilience and social support (Dumont & Provost, 1999; Rouse, 2001), and this indicates that the studies conducted in this area were not comprehensive, and each of the researchers has been separately investigating a specific domain according to their scientific background.

Hemenover (2003) pointed to behavioural factors, in that when people become aware of their emotional status in different situations, managing and controlling their emotions become easier. This is in line with the extracted codes of self-control and other-control of children in disasters that control the behaviour of the children themselves and of others in order to help the community to return to normal conditions. Furthermore, according to the results of this study on behavioural skills, we can point to voluntarily helping by children and their taking commands without any expectations. One of behavioural capacities of children is that they will undertake any given task within their capabilities and we can be sure that the task will be done. This is especially valuable in disaster situations, in which many adults do not follow commands.

Another important issue that affects children’s behavioural resilience is their availability and commitment to their friends, as they tend to enter into social activities with their peers. The present study has addressed behavioural skills and group activities. Other research has also referred to the undeniable role of friends and peers in the lives of teenagers. The results of other studies show that friends influence the personal and psychological characteristics of adolescents, facilitate their social adaptation (Sallquist, DiDonato, Hanish, Martin, & Fabes, 2012), and promote their welfare and well-being (Gross-Manos, 2014).

However, it should be noted that the lifestyle of families and societies is changing, such that, although the growth of technology and the age of information and communication have strengthened some areas of children’s capacity, this process has also reduced the physical and emotional capacities of children over time. The development of children’s physical skills, unlike their mental and intellectual development, is poorer than in the past, and this threatens the health of children and adolescents in the twenty-first century. With the children’s help, they should be encouraged towards adopting healthier behaviour and leading a more active life.

Another finding of this study was the nature of resilience, which was found to be both intrinsic and extrinsic. Although some experts have insisted on the intrinsic nature and others on the external nature of resilience, they have generally acknowledged that each individual has a proportion of resilience in him/herself, and by interacting with the environment and participating in social activities, can promote his/her behavioural, social, and psychological components and ultimately become more resilient. In line with the results of this study, Southwick, Douglas-Palumberi, and Pietrzak (2014) believed that resilience exists in all individuals on a minimum to maximum spectrum and to various degrees, and can show itself in different situations and periods. As some studies have indicated, resilience is a personal and internal characteristic that exists in all individuals to a some degree and moderates the negative effects of stressful events (Neill & Dias, 2001; Southwick et al., 2014; Wagnild & Young, 1993). Resilience can lead to personal growth, acquisition of additional resources, and adaptive skills during stressful events (Luthar, 2006; Tusaie & Dyer, 2004), and can be instilled in children and adolescents who are in their developmental stages of their life. This concept can be learned and developed in children. This shows that children should be considered in community-based actions, to which governments and policymakers are paying attention as the best response strategy to disaster, and an active role should be defined for them. Nowadays, the preferred approach is to use local people to rescue and help others, as this is both efficient and cost-effective, as shown in the above example of the social capacity of children.

### Strengths and weaknesses of the study

Despite the importance of the concept of resilience as an interactive structure, especially in disasters and accidents, no study aiming to identify and determine the components of resilience from the optimistic point of view was found in Iran or anywhere else in the world. Therefore, the current research team tried to fill this gap by determining the objective and tangible components of resilience, and directing future studies towards measurement and instrument design.

Since most studies have pointed out the vulnerability of children in disasters, this qualitative study has tried to look at the issue from a new angle, with an optimistic point of view. Although the study tried to address children’s resilience in disasters comprehensively, since the cultural, social, and climatic contexts can affect the components of resilience, it was not able to address ethnic differences. Gaining access to experts was another limitation in this study, but interviews were conducted with renowned experts in major international and national congresses.

It is essential to evaluate the components of resilience using a cross-sectional study design, so this research could form the basis for other related studies in the future.

## Conclusion

Children’s resilience in natural disasters, with a positive psychological approach, indicated two categories, comprising internal and external components. These components included psychological, emotional, cognitive, mental, spiritual, and physical components, as well as social and behavioural skills that directly and indirectly affected children’s resilience. Promoting these components in children can increase their readiness in times of disaster and reduce their vulnerability. However, for the comparison of components, quantitative studies are needed in the future to assess the extent of children’s resilience in disasters. The findings of this study showed that, as children’s resilience is a multidimensional concept, different specialities are required for the study of this concept. Moreover, to assess children’s and adolescents’ resilience in different age groups, the use of specialized teams is needed. Resilience depends on the cultural context and the extracted components in this study are applicable in Iranian society; therefore, other societies may have different components based on the opinions of different experts.
